# Integrated analysis and experimental validation of E2F2 as a potential prognostic biomarker and its oncogenic roles in serous ovarian cancer

**DOI:** 10.3389/fmolb.2025.1661558

**Published:** 2025-09-23

**Authors:** Fengyin Jiang, He Fei, Lina Yang, Rujun Chen, Liwen Zhang

**Affiliations:** Department of Gynecology & Obstetrics, Shanghai Fifth People’s Hospital, Fudan University, Shanghai, China

**Keywords:** serous ovarian cancer, E2F transcription factor 2, tumor microenvironment, chemotherapy resistance, cell-cycle regulator

## Abstract

**Background:**

This study evaluated the prognostic role of E2F transcription factor 2 (E2F2) in serous ovarian cancers (SOCs) and explored its biological functions, immune cell infiltration links, and therapeutic implications.

**Methods:**

Integrating TCGA/Genotype-Tissue Expression (GTEx) data, we used bioinformatics tools (ssGSEA, Immunophenoscore, and oncoPredict) to analyze pathways and treatment responses. Validation involved RT-qPCR, Western blot analysis, cytotoxicity, and transwell assays.

**Results:**

E2F2 was upregulated in SOC tumors, correlating with poorer overall/disease-free survival and higher tumor grade. Five cell-cycle-related genes (*ORC1*, *RAD54L*, *CCNF*, *NCAPH*, and *HASPIN*) showed strong co-expression. A pathway analysis of 808 differentially expressed genes linked E2F2 to immune cell recruitment, including CD4^+^ T cells, NK cells, and Tregs; low E2F2 levels were associated with higher immune scores. High E2F2 predicted sensitivity to chemotherapy/targeted therapy, while low E2F2 correlated with anti-CTLA4 responsiveness. *In vitro*, E2F2 promoted metastasis.

**Conclusion:**

High E2F2 expression marks poor prognosis and immune cell infiltration in SOCs and thus acts as an independent risk factor. It may serve as a potential biomarker for diagnosis, patient stratification, and guiding personalized therapy. Further research could enhance SOC management.

## Introduction

Ovarian cancer is one of the most prevalent gynecological conditions. Globally, approximately 200,000 women are diagnosed with ovarian cancer annually (Torre et al., 2018). Serous ovarian cancer (SOC), the most common subtype, is distinguished by its aggressive behavior (Li Y. et al., 2021). Despite ongoing research into SOC’s pathology and molecular genetics, its 5-year survival rate has shown minimal improvement (Matulonis et al., 2016). This is largely attributable to SOC’s asymptomatic presentation in its early stages and the lack of reliable biomarkers, leading to most cases being diagnosed at intermediate or advanced stages. Thus, elucidating the molecular mechanisms underlying SOC and identifying reliable biomarkers are critical for improving patient outcomes.

E2F transcription factor 2 (E2F2) is an established key regulator of the cell cycle in malignant tumors. Conventional understanding identifies it as an essential gene in this regulatory process (Shen and Wang, 2021; Du et al., 2022; Li et al., 2018). In ovarian cancer, E2F2 is currently regarded as an oncogenic gene associated with a poor prognosis. An analysis of 308 ovarian cancer samples demonstrated that E2F2 expression is significantly upregulated in ovarian cancer epithelial cells and correlates with reduced overall survival (Xie et al., 2017). Among 77 ovarian cancer samples, all E2F transcription factors except E2F6 showed significant overexpression relative to control samples, with E2F2 exhibiting the highest mRNA levels. We found that elevated E2F1, E2F2, and E2F8 levels were strongly linked to histopathological grade 3 ovarian tumors and residual lesions ≥2 cm post primary tumor debulking surgery. In *in vitro* studies, all three E2Fs were upregulated in ovarian cancer cell lines compared with human peritoneal mesothelial cells, with E2F2 showing the most dramatic increase (up to 30-fold). These results suggest that E2F2 may play a more pivotal role in ovarian cancer biology than E2F1. Thus, E2F2 holds promise as a predictive biomarker for ovarian cancer and could inform novel therapeutic strategies.

Nevertheless, the molecular mechanisms underlying E2F2-mediated regulation of ovarian cancer remain incompletely understood. Xie et al. (2017) demonstrated that elevated E2F2 expression significantly enhances the transcription of MCM4, CCNE2, and WHSC1 in SKOV3 and A2780 ovarian cancer cell lines—all of which possess oncogenic properties. Additionally, LBX2-AS1, a novel long non-coding RNA (lncRNA) that promotes ovarian cancer progression, exerts its effects by regulating E2F2 expression; notably, high E2F2 expression can counteract the effects of LBX2-AS1 knockdown (Cao et al., 2021). Given the current limitations in SOC diagnosis and treatment, further investigation into E2F2’s role in SOC holds substantial promise. Elucidating E2F2’s functions in SOC may not only facilitate the development of E2F2-targeted therapies but also enable identification of biomarkers for early detection and accurate prognostic prediction, thereby advancing the clinical management of SOC.

Accumulating recent evidence indicates that E2F2 functions as a key regulator of cell-cycle progression, apoptosis (Zhao et al., 2017), and inflammation (Zhang et al., 2018). These findings challenge conventional perspectives and underscore the intricate role of E2F2 within the tumor microenvironment (TME). Like most solid tumors, ovarian tumors exhibit immunogenicity (Garsed et al., 2022); however, whether E2F2 contributes to TME formation in SOC or correlates with therapeutic responses in SOC remains unknown.

The present study aimed to evaluate E2F2 expression levels and prognostic significance in SOC using bioinformatics databases. Gene set enrichment analysis was employed to identify functional roles, genetic alterations, and pathways within the E2F2 regulatory network associated with SOC. Additionally, we analyzed the association between E2F2 and tumor-infiltrating immune cells. Furthermore, we assessed E2F2’s role in immune responses to elucidate its significance in SOC pathogenesis.

## Materials and methods

### Datasets acquisition and processing

We retrieved expression profiles along with clinical information for 378 serous ovarian carcinoma (SOC) patients from TCGA database (https://portal.gdc.cancer.gov/). The key clinical characteristics of this TCGA dataset are shown in Table 1. The expression patterns of normal tissues including 180 samples were procured from the Genotype-Tissue Expression (GTEx) project (https://gtexportal.org/home/). To establish the prognostic significance of E2F2 in SOCs, data were obtained from the GSE9891, GSE63885, GSE26193, and GSE18520 datasets. Single-nucleotide variant (SNV) data and copy number variations (CNV) of E2F2 in SOCs were downloaded from cBioPortal (https://www.cbioportal.org/). The databases and sample information involved in this study are shown in Table 2.

**TABLE 1:** Key clinical characteristics of TCGA cohorts used.

Characteristic	Low expression of E2F2	High expression of E2F2	P value
N	189	189	
Clinical stage, n (%)			0.251
Stages I and II	8 (4%)	15 (8%)	
Stage III	148 (79%)	146 (78%)	
Stage IV	32 (17%)	26 (14%)	
Tumor status, n (%)			0.961
Tumor-free	36 (21%)	35 (21%)	
With tumor	133 (79%)	131 (79%)	
Race, n (%)			0.537
Asian	5 (3%)	6 (3%)	
Black or African American	15 (8%)	10 (6%)	
White	160 (89%)	168 (91%)	
Venous invasion, n (%)			0.128
No	22 (47%)	18 (32%)	
Yes	25 (53%)	38 (68%)	
Lymphatic invasion, n (%)			0.352
No	26 (36%)	22 (29%)	
Yes	46 (64%)	54 (71%)	
PFI event, n (%)			0.729
No	50 (26%)	53 (28%)	
Yes	139 (74%)	136 (72%)	

Note: Data for “Venous invasion” and “Lymphatic invasion” are based on available samples with complete records.

**TABLE 2:** Databases and sample information.

Database name	URL	Sample type	Sample size	Access date
TCGA	https://portal.gdc.cancer.gov/	Ovarian cancer	378	2024.05
GTEx	https://gtexportal.org/home/	Normal ovarian epithelial tissue	180	2024.05
GEO	https://www.ncbi.nlm.nih.gov/geo/	Ovarian cancer	520	2024.05
cBioPortal	https://www.cbioportal.org/	Ovarian cancer	378	2024.05
Kaplan-Meier plotter	https://kmplot.com/analysis/	Ovarian cancer	520	2024.05
GEPIA2	http://gepia2.cancer-pku.cn/	Ovarian cancer	378	2024.05

### Expression level and prognostic value

To analyze the disparity in E2F2 expression across groups, we employed the Wilcoxon rank-sum test. To evaluate the prognostic implications of E2F2 in SOCs within TCGA and GEO datasets, the Kaplan–Meier method was implemented via the Kaplan-Meier plotter, available at https://kmplot.com/analysis/. The genetic mutation landscape for E2F2 was generated using the R package “maftools.” All statistical inferences considered a p-value below 0.05 as indicating statistical significance.

### Co-expressed genes

We determined genes co-expressed with E2F2 by setting corFilter at 0.3 and *P* at 0.001 as threshold values. To create visual representations of the findings, the “circlize” R package was utilized.

### Functional enrichment analysis

Based on the expression levels of E2F2, SOC samples were split into two distinct groups. To pinpoint differentially expressed genes (DEGs), we contrasted the expression patterns between these two groups. Using the R package “clusterProfiler,” we carried out Gene Ontology (GO) and Kyoto Encyclopedia of Genes and Genomes (KEGG) pathway analyses on the DEGs, with an enrichment cut-off of *P* < 0.05. Moreover, gene set enrichment analysis (GSEA) was executed with the “clusterProfiler” R package, leveraging the c2.cp.kegg.v7.4.symbols geneset.

### Tumor microenvironment (TME) and immune function analysis

The tumor microenvironment (TME) is composed of tumor cells, extracellular matrix (ECM), related stromal and immune cells, and signaling substances (Yang, 2017). We calculated the stromal, immune, and ESTIMATE scores of E2F2 by applying the ESTIMATE algorithm (Yoshihara et al., 2013). This algorithm facilitated a breakdown of the E2F2 scores related to the different cellular components of the TME, highlighting the scores associated with stromal cells, immune cells, and the overall ESTIMATE score, which reflects the relative abundance of stromal and immune cells in the TME. After estimating the infiltration of immune cells, we carried out Spearman correlation analysis. This assessed the connection between E2F2 expression and immune cell quantity, making use of seven immune-related databases: xCell, TIMER, quanTIseq, MCPcounter, EPiC, CIBERSORT-ABS, and CIBERSORT. Additionally, Spearman correlation analysis was performed to explore the link between E2F2 expression and immune checkpoints. To display the results, we utilized the “corrplot” R package.

### E2F2-based treatment strategy

The Immunophenoscore (IPS) from the TCIA dataset was used to evaluate the role of E2F2 in the immune response (Charoentong et al., 2017). These scores aid physicians in identifying patients better suited for immune checkpoint therapies. Drug sensitivity to standard chemotherapy and targeted therapies was predicted using the OncoPredict R package. Drug sensitivity was determined by measuring the half maximal inhibitory concentration (IC_50_) of drugs; a high IC_50_ indicates low sensitivity.

### Cell culture and transfection

The HeyA8 and A2780 human ovarian epithelial cancer cell lines were provided by American Type Culture Collection (ATCC). These cells were cultivated in RPMI 1640 medium, supplemented with 10% fetal bovine serum. The incubation occurred within an environment maintained at 37 °C and a humidified 5% CO_2_ atmosphere. Lentiviral vectors for E2F2 overexpression and their corresponding negative controls were purchased from Shanghai Genechem Company (Shanghai, China). Two short hairpin RNAs (shRNAs) targeting E2F2 were cloned into the pLKO.1 vector and synthesized by Sangon Biotech (Shanghai, China). In 6-cm dishes, 293T cells at 80% confluence were transfected with E2F2 overexpression or shRNA plasmids, their respective negative controls, pSPAX2, and pVSVG plasmids in a 4:3:1 μg mass ratio using Lipofectamine 3000 as per the manufacturer’s instructions. Viral supernatants were harvested 48 h and 72 h after transfection to infect HeyA8 and A2780 cells. After 48 h, the medium was substituted with standard growth medium supplemented with puromycin (2 μg/mL) for a 7-day selection period.

### Reverse transcription quantitative PCR (RT-qPCR)

After discarding the culture medium, the cells in 6-well plates underwent two washes with 1× PBS. RNA isolation was carried out using 1 mL of TRIzol reagent. The lysate was transferred to a 1.5-mL Eppendorf tube, and then 200 μL of trichloromethane was introduced into the tube. The mixture was placed on ice for 3 min and then centrifuged at 12,000 rpm for 10 min at 4 °C. The aqueous layer was carefully retrieved, and RNA precipitation was achieved with isopropanol and 75% ethanol. To generate complementary DNA (cDNA) from the isolated RNA, the Takara RT reagent kit (#RR047A, Takara, China) was employed. Sangon Biotech in Shanghai, China, synthesized the primers. RT-qPCR was performed on a QuantStudio 6 instrument using SYBR Green (#A25741, Thermo Fisher Scientific, USA). The comparative CT method (2^−ΔΔCT^) was used to measure gene expression levels. The RT-qPCR primers are listed in Table 3.

**TABLE 3:** Primers used in the study.

Gene	Sequence (5′-3′)	Application
*ORC1*-F	ACCGAGATTCACATCCAGATTGG	RT-qPCR
*ORC1*-R	CGAGCACGTTTCTTAGGAGGA	RT-qPCR
*RAD54L*-F	TTGAGTCAGCTAACCAATCAACC	RT-qPCR
*RAD54L*-R	GGAGGCTCATACAGAACCAAGG	RT-qPCR
*CCNF*-F	CACAAAGCATCCATATTGCACTG	RT-qPCR
*CCNF*-R	TGGTCAGACATCCCTGATGAG	RT-qPCR
*NCAPH*-F	GTCCTCGAAGACTTTCCTCAGA	RT-qPCR
*NCAPH*-R	TGAAATGTCAATACTCCTGCTGG	RT-qPCR
*HASPIN*-F	ACAGTGTCATCTCGATCGGC	RT-qPCR
*HASPIN*-R	GACCATCCTGGTGTCCTTGG	RT-qPCR
*E2F2*-F	CGTCCCTGAGTTCCCAACC	RT-qPCR
*E2F2*-R	GCGAAGTGTCATACCGAGTCTT	RT-qPCR

### Western blot analysis

Protein concentration was determined using the BCA assay, and concentrations across samples were normalized with 5× protein loading buffer and RIPA to ensure equal loading; samples were boiled at 100 °C for 5–10 min and stored at −80 °C. For electrophoresis, 10–30 μg of protein samples was loaded onto 10% SDS-PAGE gels and run at 80 V for 30 min and then 120 V for 90 min for sufficient separation. Polyacrylamide gels were transferred onto PVDF membranes with filter papers and sponges (air bubbles expelled by rolling), followed by transfer at 90 V for 2 h on ice using a transfer apparatus. PVDF membranes were blocked in TBST containing 5% non-fat milk for 1 h at room temperature, washed twice with TBST (5 min each), then incubated with E2F2 antibody (#CY5805, Abways, China) against the target protein on a shaker at 4 °C overnight. After three 10-min washes with TBST, membranes were incubated with corresponding secondary antibodies on a shaker at room temperature for 2 h, followed by three 10-min TBST washes. Finally, membranes were visualized using ECL ultra-sensitive luminescent solution, with images captured via a Tanon 5200 gel imaging system.

### Cell cytotoxicity assays

We plated HeyA8 and A2780 ovarian cancer cells into 96-well plates at a density of 8,000 cells per well. The plates were transferred to a 37 °C humidified incubator with a 5% CO_2_ environment. To ensure proper cell attachment, we incubated them overnight. Cisplatin was added at concentrations of 0, 2.5, 10, 40, 160, and 640 μM (for paclitaxel, the concentrations were 0, 1, 5, 25, 125, and 625 nM). Once the 48-h treatment concluded, 10% CCK-8 solution was added to every well of the plate. The plate underwent incubation for a set time, enabling the generation of formazan crystals. After that, a NanoQuant plate reader was used to measure the optical density (OD) at 450 nm. Cell viability was determined using the formula “cell viability = (OD of treated group − OD of blank)/(OD of control group − OD of blank).”

### Transwell assays

The upper chamber of a 24-well transwell plate (Corning #3422, USA) was coated with 60 μL of Matrigel, diluted 1:8 in serum-free RPMI 1640, and incubated at 37 °C for 1–3 h. A volume of 500 μL of RPMI 1640 culture medium, fortified with 10% FBS, was carefully pipetted into the lower chamber. HeyA8 (100 μL, 1 × 104) or A2780 (100 μL, 1.5 × 104) cells were placed in the upper chamber and incubated for 35 h (46 h for A2780). Non-invasive cells were removed from the upper chamber. The chamber was treated with anhydrous methanol for 20 min, stained using 0.1% crystal violet for 30 min, and subsequently photographed. For migration assays, the upper chamber was not coated with Matrigel, and the cell number was doubled compared to the invasion assay, with other steps remaining unchanged. Finally, we photographed migrating or invading cells under a Nikon high-resolution microscope at ×10 magnification, and the cell count for each group was the average of counts from five random fields of view.

### Statistical analysis

Differences between continuous variables were assessed using either the Wilcoxon rank-sum test or Student’s t test. Correlation between two continuous variables was analyzed using Pearson’s or Spearman’s rank correlation methods. Differences among Kaplan–Meier survival curves were constructed using a two-sided log-rank test. For all statistical analyses, a p-value threshold of less than 0.05 was adopted.

## Results

### E2F2 expression and prognostic significance in SOCs

**FIGURE 1:**
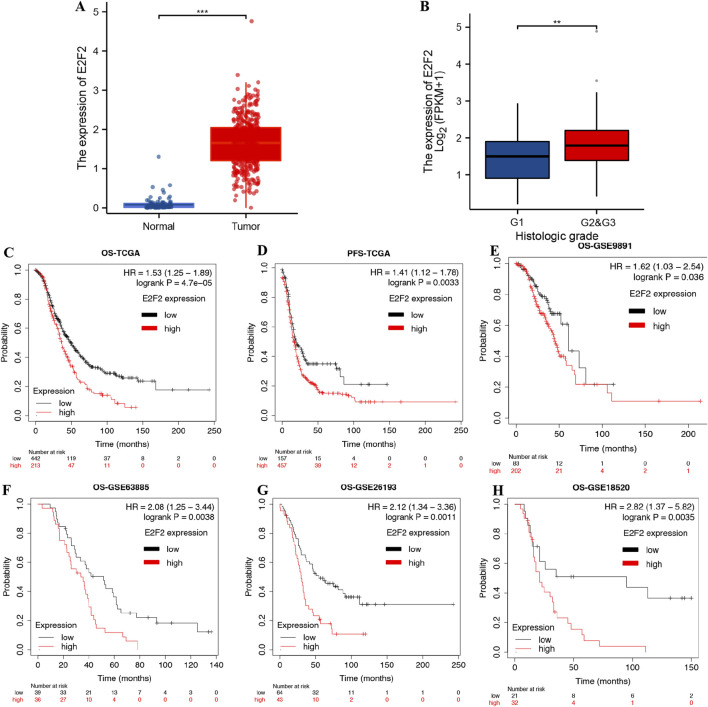
Expression and prognostic analysis of E2F2 in SOCs. **(A)** Expression of E2F2 was significantly upregulated in SOC tissues. **(B)** E2F2 expression was increased in the grade 2/3 group compared with the grade 1 group. **(C–H)** SOC patients with high expression of E2F2 had poor OS and PFS. *, *P* < 0.05; **, *P* < 0.01; ***, *P* < 0.001.

As shown in Figure 1A, E2F2 expression in serous ovarian carcinoma tissues is markedly higher than that in normal tissues (*P* < 0.001). We further investigated the correlation between E2F2 expression and clinical characteristics. Intriguingly, E2F2 expression was notably higher in the grade 2/3 SOC group than in the grade 1 group (Figure 1B, *P* < 0.01). The Kaplan–Meier method was utilized to evaluate the prognostic significance of E2F2 in SOCs by examining its correlation with overall survival (OS) and progression-free survival (PFS) in TCGA and GEO cohorts. In TCGA dataset, SOC patients with high E2F2 expression exhibited significantly lower OS (Figure 1C, *P* = 4.7e-5) and PFS (Figure 1D, *P* = 0.0033) rates than those with low E2F2 expression. High E2F2 expression correlated with poor clinical outcomes in OS analysis across the GSE9891, GSE63885, GSE26193, and GSE18520 datasets (Figures 1E–H, all *P* < 0.05). To explore whether E2F2 acts as an independent prognostic indicator, traditional clinicopathological variables and E2F2 protein expression levels were analyzed via Cox’s hazard regression model. Univariate analyses indicated that the TNM stage and high E2F2 expression were notably linked to overall survival in SOC patients (Supplementary Figure 1). Further multivariate analyses demonstrated that, alongside the TNM stage (hazard ratio (HR) 1.628, 95% confidence interval (CI) 1.410−1.878, *P* < 0.001), high E2F2 expression (HR 1.390, 95% CI 1.139−1.639, *P* < 0.001) also served as an independent prognostic factor for overall survival in SOC patients (Supplementary Figure 2). Collectively, our research has uncovered an important link between E2F2 and SOC. Given the observed associations, it is highly likely that E2F2 actively promotes SOC tumor progression. Even more significantly, our findings firmly indicate that E2F2 could potentially serve as a key prognostic biomarker.

### Genetic alterations of E2F2 in SOCs

**FIGURE 2:**
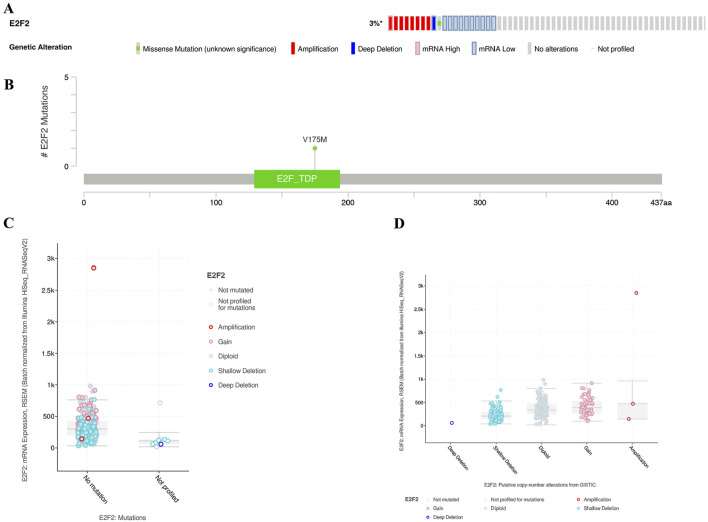
Mutation landscape of E2F2 in SOCs. **(A)** Mutation status of E2F2 in SOCs. **(B)** Location of E2F2 mutations and associated altered proteins in SOCs. Correlation between E2F2 expression and single-nucleotide variation **(C)** as well as copy number variation **(D)**. *, *P* < 0.05; **, *P* < 0.01; ***, *P* < 0.001.

Approximately 3% of SOC cases harbored an E2F2 mutation. Among the genetic alteration subtypes, amplification ranked first (Figure 2A). Figure 2B depicts the location of E2F2 mutations and associated protein changes. Figures 2C,D, respectively show the correlations between E2F2 expression and single-nucleotide variants, as well as copy number variation.

### The co-expressed genes of E2F2

**FIGURE 3:**
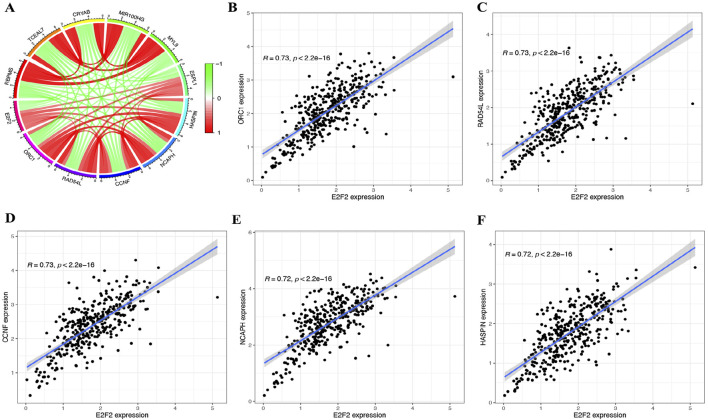
Co-expressed genes of E2F2 in SOCs. **(A)** Top six genes most positively or negatively correlated with E2F2 in SOCs. Top five genes most positively correlated with E2F2: *ORC1* **(B)**, *RAD54L* **(C)**, *CCNF* **(D)**, *NCAPH* **(E)**, and *HASPIN* **(F)**. *, *P* < 0.05; **, *P* < 0.01; ***, *P* < 0.001.

In SOCs, E2F2 showed a positive correlation (Cor >0.3, *P* < 0.001) with 1,786 genes and a negative correlation (Cor <−0.3, *P* < 0.001) with 784 genes. Figure 3A illustrates the six genes exhibiting the strongest positive or negative correlations with E2F2. The top five most positively correlated with E2F2 were *ORC1* (Cor = 0.73), *RAD54L* (Cor = 0.73), *CCNF* (Cor = 0.73), *NCAPH* (Cor = 0.73), and *HASPIN* (Cor = 0.73) (Figures 3B–F).

### E2F2-related biological functions

**FIGURE 4:**
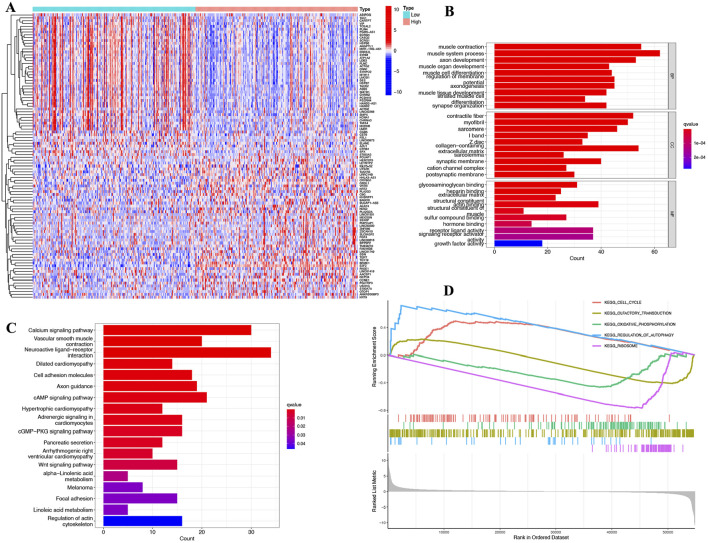
Functional and pathway enrichment analysis of E2F2-related genes. **(A)** 808 differentially expressed genes (DEGs) and expression heat maps based on E2F2 expression. **(B)** GO functional enrichment analysis of relevant biological process, cellular components, and molecular functions of interacting genes of E2F2. **(C)** KEGG pathway analysis of relevant signal pathways of interacting genes of E2F2. **(D)** GSEA of relevant biological process of DEGs. DEGs, differentially expressed genes; GO, gene ontology; KEGG, Kyoto Encyclopedia of Genes and Genomes; GSEA, Gene set enrichment analysis.

We classified SOC samples into two distinct groups based on the expression levels of E2F2. When we contrasted the expression patterns of these two groups, 808 differentially expressed genes (DEGs) were identified (|logFC| ≥2, adj.*P* < 0.05). Figure 4A presents the expression heatmap of these DEGs. Using Gene Ontology (GO) analysis, it was determined that these DEGs were predominantly associated with muscle contraction, axon development, membrane regulation, glycosaminoglycan binding, receptor ligand activity, and signaling receptor activator activity (Figure 4B). KEGG pathway analysis revealed that these DEGs are associated with the calcium signaling pathway, cell adhesion molecules, cMAP signaling pathway, cGMP–PKG signaling pathway, and Wnt signaling pathway (Figure 4C). Gene set enrichment analysis (GSEA) showed that the DEGs were primarily linked to the cell cycle, oxidative phosphorylation, and ribosomes (Figure 4D).

### Association with tumor microenvironment (TME) and immune cell infiltration

**FIGURE 5:**
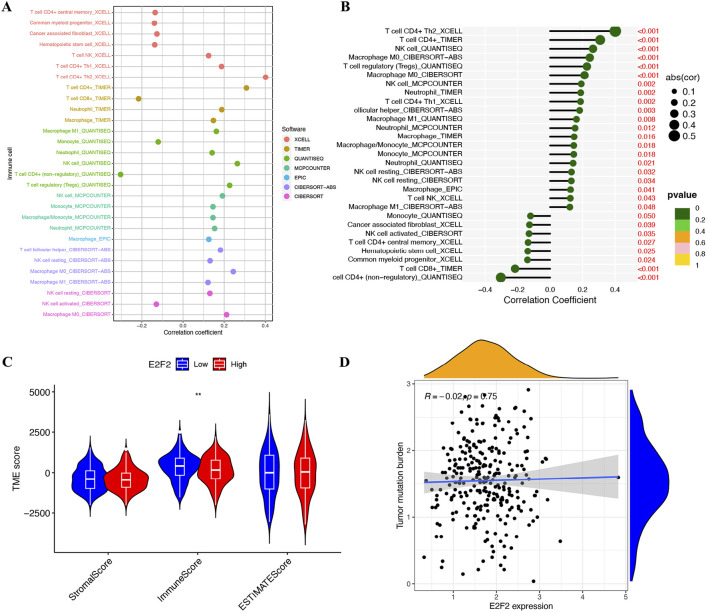
Correlation of E2F2 with tumor microenvironment and immune function. **(A,B)** Correlation of E2F2 expression with immune cells. **(C)** SOC patients with low E2F2 expression had high immuneScore. **(D)** Correlation between E2F2 and tumor mutation burden in SOCs. *, *P* < 0.05; **, *P* < 0.01; ***, *P* < 0.001.

Cancer immunology research has underscored that immune cell infiltration within tumors represents a fundamental aspect of the tumor microenvironment (TME). This infiltration exerts a profound influence on the progression of SOC. To comprehensively explore the relationship between E2F2 and immune cell quantity, we conducted an in-depth analysis using seven well-established immune-related databases: xCell, TIMER, quanTIseq, MCPcounter, EPiC, CIBERSORT-ABS, and CIBERSORT. As shown in Figures 5A,B, E2F2 expression was positively associated with most types of immune cells. Specifically, E2F2 expression levels were directly proportional to the quantity of CD4^+^ T cells, NK cells, M0 macrophages, Tregs, and neutrophils, as clearly illustrated in Figure 5B. Moreover, low E2F2 expression was associated with high immunoreactivity (Figure 5C). E2F2 expression showed no significant correlation with tumor mutation burden in SOCs (Figure 5D).

### Correlation with immune checkpoints and treatment response

**FIGURE 6:**
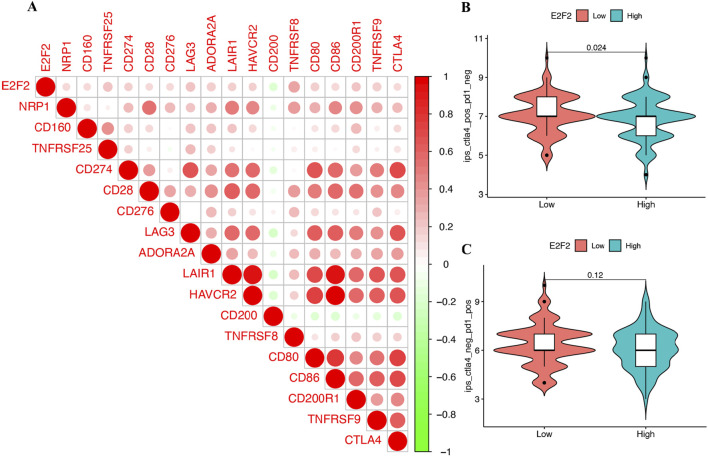
Treatment strategy for SOCs based on E2F2. **(A)** Correlation between E2F2 expression and immune checkpoints. **(B)** SOC patients with low E2F2 expression responded better in anti-CTLA4 treatment. **(C)** No significant difference in anti-PD1 treatment between patients with low E2F2 expression and patients with high E2F2 expression of SOC. *, *P* < 0.05; **, *P* < 0.01; ***, *P* < 0.001.

To explore the role of E2F2 in guiding SOC therapy, we analyzed its correlation with immune checkpoints. Statistical analysis found a strong positive link between the expression levels of E2F2 and the expression of immune checkpoints like PD1 and CTLA4 (Figure 6A). Immunophenoscore (IPS) analysis revealed that SOC patients exhibiting low E2F2 expression responded more favorably to anti-CTLA4 treatment than those with high E2F2 expression (Figure 6B). However, in our study, all groups showed a similar response to anti-PD1 therapy. After categorizing patients into distinct groups based on specific criteria, we administered anti-PD1 treatment. However, comprehensive evaluation showed no significant divergence in treatment outcomes between these groups (Figure 6C).

### Estimation of therapeutic agent sensitivity

**FIGURE 7:**
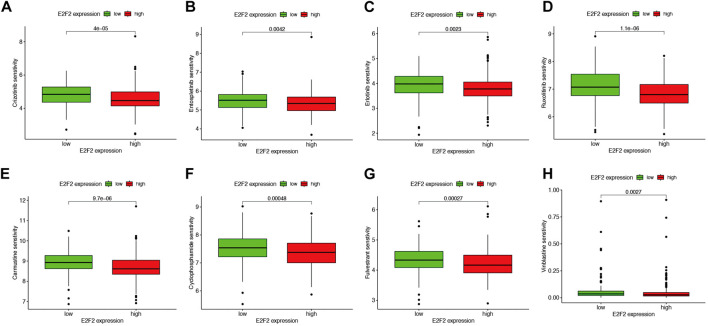
Correlation between E2F2 expression and common drugs for chemotherapy and targeted therapy. Average of IC_50_ score of crizotinib **(A)**, entospletinib **(B)**, erlotinib **(C)**, ruxolitinib **(D)**, carmustine **(E)**, cyclophosphamide **(F)**, fulvestrant **(G)**, and vinblastine **(H)** in the high E2F2 expression group decreased versus that in the low E2F2 expression group. *, *P* < 0.05; **, *P* < 0.01; ***, *P* < 0.001.

The oncoPredict R package was used to analyze the association between E2F2 expression and drug sensitivity (IC_50_) to chemotherapy and targeted therapy drugs. The study indicated that drugs, including crizotinib, tospletinib, erlotinib, ruxolitinib, carmustine, cyclophosphamide, fulvestrant, and vinblastine, exhibited significantly reduced IC_50_ values in the group with high E2F2 expression contrasted with the group with low E2F2 expression (Figures 7A–H, all P < 0.05), indicating increased drug sensitivity in the high E2F2 expression group. The IC_50_ values for platinum and paclitaxel were comparable between the two groups (data not shown). These findings suggest that E2F2 expression levels could potentially serve as an auxiliary basis for SOC treatment decisions.

### Experimental validation of E2F2 function in SOC

**FIGURE 8:**
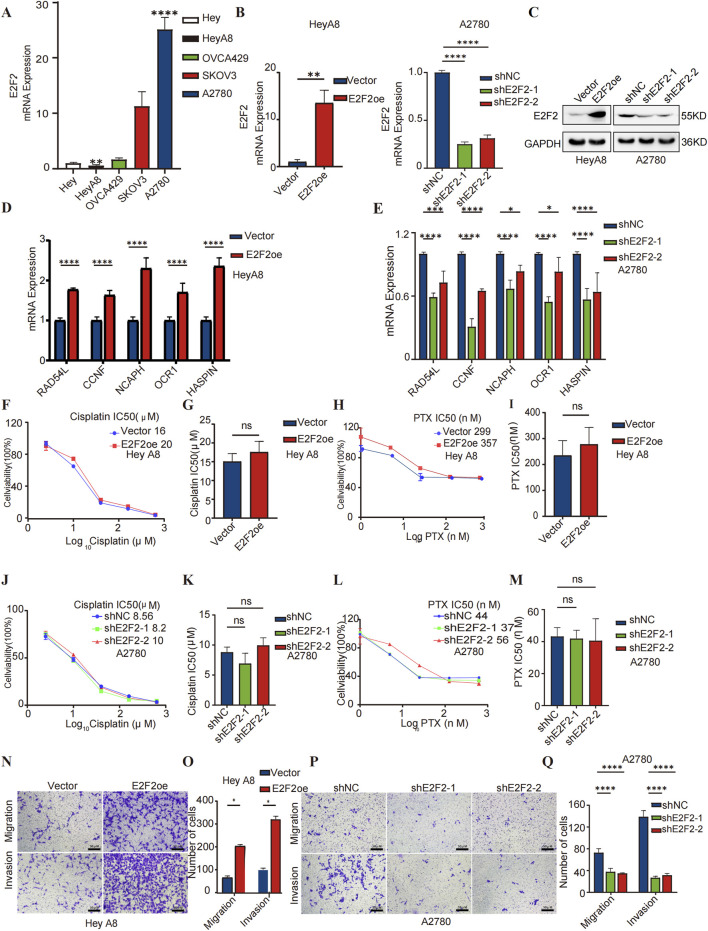
Experimental validation of E2F2 function in SOC. **(A)** Detection of E2F2 mRNA levels in five ovarian cancer cell lines (Hey, HeyA8, OVCA429, SKOV3, and A2780) by RT-qPCR. GAPDH was used to normalize the expression of E2F2. **(B)** RT-qPCR analysis showing the efficiency of E2F2 overexpression in HeyA8 cells and E2F2 knockdown in A2780 cells. **(C)** Western blot analysis demonstrating the efficacy of E2F2 overexpression in HeyA8 cells and E2F2 knockdown in A2780 cells. **(D,E)** RAD54L, CCNF, NCAPH, ORC1, and HASPIN mRNA expression of E2F2-overexpression HeyA8 or E2F2-knocking down A2780 and the respective negative control cell. **(F–M)** Paclitaxel or cisplatin IC_50_ and viability comparison between E2F2-overexpression HeyA8 or E2F2-knocking down A2780 and respective negative control cell. **(N,P)** Representative images analysis of Transwell migration/invasion assays using E2F2-overexpression HEY A8 cells and E2F2-knockdown A2780 cells, Scale bars = 50 μm. **(O,Q)** Quantitative analysis of migration and invasion cells (P < 0.05). Error bars = 95% CIs. All dates presented as mean ± SD, n = 3 biologically independent repeats.*, *P* < 0.05; **, *P* < 0.01; ***, *P* < 0.001; ****,*P* < 0.0001; ns, not statistically significant.

The expression level of E2F2 mRNA in five human ovarian epithelial cancer cell lines was evaluated using RT-qPCR. The results showed that A2780 cells had the highest E2F2 expression level, while HeyA8 cells had the lowest (Figure 8A). Subsequently, HeyA8-E2F2oe cells stably expressing E2F2 cDNA, A2780-shE2F2-1/2 cells with E2F2 knockdown, and their respective negative control cells—all validated at both the mRNA and protein levels—were established (Figures 8B,C).

RT-qPCR was employed to verify the co-expression of E2F2 with five other genes identified via bioinformatics by measuring the expression levels of these mRNAs in HeyA8-E2F2oe, A2780-shE2F2, and their control cells. The expression of *RAD54L*, *CCNF*, *NCAPH*, *ORC1*, and *HASPIN* genes was consistent with E2F2 expression, whether in A8 cells with high E2F2 expression and their negative controls or in A2780 cells with E2F2 knockdown and their negative controls (Figures 8D,E).

Given that platinum and paclitaxel constitute the standard first-line adjuvant treatment for high-grade serous ovarian carcinoma (HGSOC) (Konstantinopoulos and Matulonis, 2023), we conducted a cytotoxicity assay across six drug concentrations to further validate E2F2’s role in response to paclitaxel and cisplatin. As shown in Figures 8F–I, HeyA8 cells overexpressing E2F2 did not exhibit significant differences in response to cisplatin or paclitaxel compared to the control group. The same was true for A2780 cells with E2F2 knockdown (Figures 8J–M). These experimental results were consistent with the bioinformatics analysis in the E2F2-based treatment strategy for SOCs section.

The impact of E2F2 on cancer cell metastasis was examined using a transwell assay. The findings indicated that E2F2 overexpression increased the migration and invasion abilities of HeyA8 cells relative to the negative control group, whereas E2F2 inhibition reduced cell metastasis (Figures 8N–Q). *In vitro* experiments indicated a positive correlation between E2F2 expression and poor SOC progression, supporting E2F2 as a potential prognostic marker in SOC.

## Discussion

Ovarian cancer has a high mortality rate, primarily due to late-stage detection; thus, early diagnosis is critical. Beyond classic clinical biomarkers such as CA125 and HE4, recent studies have uncovered novel candidates in ovarian cancer. For example, Hong et al. (2024) were the first to systematically elucidate the oncogenic role of IRF6 in ovarian cancer, proposing it as a potential diagnostic biomarker and therapeutic target. Our study demonstrated significant E2F2 upregulation in serous ovarian carcinoma (SOC) tissues relative to normal tissues, with notably higher expression in grade 3/4 SOC. Kaplan–Meier analyses further identified E2F2 as a potential prognostic factor in SOC. We additionally characterized E2F2 expression in SOC, including its involvement in pathway crosstalk, immune cell infiltration, and implications for therapeutic guidance.

Despite being discovered 35 years ago, E2F2 remains enigmatic. Its roles are extensively studied in various disciplines, including biochemistry, cell and developmental biology, and oncology (Li L. et al., 2021). Recent studies suggest that E2F2 may either inhibit or promote cell proliferation, contingent upon the cellular environment. In normal dividing progenitor cells, E2F2 functions as a transcriptional activator, which is essential for cell survival. Our research demonstrated that E2F2 was co-expressed with *ORC1*, *RAD54L*, *CCNF*, *NCAPH*, and *HASPIN*. These genes are functionally associated with DNA replication, synthesis, and chromosomal activities (Jaremko et al., 2020; Machida et al., 2014; Martin et al., 2016; Zhou et al., 2014). Previous studies have shown that the E2F family exhibits highly complex regulatory relationships with *RAD54L*, *ORC1*, *CCNF*, and other genes. For instance, ChIP-chip assays by Ren et al. (2002) demonstrated that E2F4 is enriched at the RAD54L promoter region in quiescent (G0) human primary fibroblasts (WI-38). E2F1 also binds to the same promoter during the G1/S transition, indicating that RAD54L is a dual target gene of E2Fs (with E2F4-mediated repression and E2F1-mediated activation). Additionally, they verified that the ORC1 promoter is co-bound by both E2F4 and E2F1, suggesting that E2Fs directly regulate ORC1 transcription, ensuring the synchronization of replication initiation with the cell cycle (Ren et al., 2002). Linda Clijsters and colleagues identified that cyclin F (CCNF) acts as a specific “temporal switch” for activated E2F1/2/3A during the late S/G2 phase: by recognizing their N-terminal CY motif and mediating proteasomal degradation, CCNF terminates E2F-driven transcriptional programs, thereby preventing cell-cycle dysregulation and genomic instability (Clijsters et al., 2019). Our bioinformatics analysis and experimental validation primarily focus on the expression relationships between E2F2 and these genes, suggesting that E2F2 may be involved in the transcription of these genes and thus participate in the regulation of cell cycle, DNA replication, and chromosomal activities. Conversely, in differentiated cells, E2F2 forms a complex with Rb and functions as a transcriptional inhibitor, facilitating the cell’s exit from the cell cycle. In cancer cells, RB is frequently inactivated. This inactivation leads to a shift in E2F2’s role from a repressor to an activator, resulting in the overactivation of E2F2 target genes and uncontrolled cell division (Chong et al., 2009). Research indicates that E2F2 generally facilitates tumor progression in various cancers (Feliciano et al., 2017; Yu et al., 2020), although there is some evidence of its potential tumor-suppressive effects (Opavsky et al., 2007). Our research detected heightened E2F2 expression in SOCs, with elevated levels linked to worse overall and progression-free survival in patients. *In vitro* analyses additionally showed a positive association between E2F2 and cancer cell metastatic ability. These findings align with earlier studies on E2F2’s tumor-promoting effects (Zhang et al., 2021; Zhou et al., 2019), indicating its potential as a novel biomarker for SOCs.

SOCs are identified as “immunogenic tumors” due to their capacity to elicit non-spontaneous anti-tumor immune responses in the tumors, peripheral blood, and ascites of patients (Santoiemma et al., 2016). Immune cells in tumors and ascites—including T and B lymphocytes, Tregs, NK cells, tumor-associated macrophages, and myeloid-derived suppressor cells—play crucial roles in the development and progression of SOCs (Rodriguez et al., 2018). Considering E2F2’s reported role in controlling immune infiltration within the colorectal cancer microenvironment (Shang et al., 2022), we examined the connection between E2F2 and the tumor microenvironment (TME) in SOCs. The research identified a positive link between E2F2 expression and the presence of CD4^+^ T cells, NK cells, M0 macrophages, Tregs, and neutrophils. Lower levels of E2F2 expression were linked with increased immunoreactivity. These findings suggest that E2F2 has a regulatory function in immune infiltration within the tumor microenvironment of SOCs.

E2F2 thus emerges as a pivotal coordinator of proliferative signaling and immune microenvironment remodeling in SOC, with our multi-database analyses (xCell, TIMER, xCell, quanTIseq, MCPcounter, xCell, EPiC, CIBERSORT-ABS, and CIBERSORT) revealing mechanistic links between these dual functions. As a canonical cell-cycle regulator, E2F2 drives G1/S transition through the transcriptional activation of cyclin E1 and CDK2, fueling the uncontrolled proliferation of ovarian epithelial cells—consistent with our observation of elevated E2F2 in 378 SOC samples and its association with reduced overall survival. Concurrently, our immune infiltration analyses demonstrate that E2F2 expression correlates positively with key immune cell populations: CD4^+^ T cells, NK cells, Tregs, M0 macrophages, and neutrophils. This immunomodulatory footprint, paired with the finding that low E2F2 expression associates with high immunoreactivity, suggests that E2F2 orchestrates a pro-tumorigenic balance between rapid cell division and immune evasion.

The intersection of these functions forms a pathogenic circuit in SOC: E2F2-mediated proliferation expands tumor mass while its induction of chemokine signaling (e.g., CXCL12 and CCL2) likely recruits immune subsets that simultaneously support tumor growth and dampen anti-tumor responses. Notably, Tregs—whose abundance correlates with E2F2 levels in our dataset—are well-documented as suppressing cytotoxic T cell activity in ovarian cancer, while M0 macrophages can polarize toward a pro-tumor M2 phenotype upon tumor microenvironment cues. This may explain why high E2F2 expression associates with both increased immune cell infiltration and reduced immunoreactivity—the recruited cells predominantly exert immunosuppressive functions, enabling proliferating tumor cells to evade immune surveillance.

High mortality in SOC is strongly associated with poor responses to therapies, including conventional chemotherapy, immunotherapy, and targeted agents (Yang et al., 2022), highlighting the urgent need for predictive biomarkers to guide treatment decisions. Current research has centered on genes linked to platinum or paclitaxel resistance; for instance, Zhu et al. (2024) demonstrated high expression of NDRG1, CYBRD1, and MT2A in cisplatin-resistant ovarian cancer cells while identifying Photofrin as a potential agent to reverse such resistance.

Our investigation into the role of E2F2 in guiding SOC therapy revealed that high E2F2 expression correlates with increased sensitivity to chemotherapy and targeted agents, whereas low E2F2 expression associates with improved responses to anti-CTLA4 therapy. Notably, E2F2 exhibits a nuanced role in therapeutic guidance; *in vitro* experiments (Figures 8F–M) showed no significant differences in cisplatin or paclitaxel sensitivity between E2F2-overexpressing, E2F2-knockdown, and control cells, which is consistent with bioinformatics analyses that demonstrate no correlation between E2F2 expression and IC50 values for these agents. This aligns with the distinct mechanisms of first-line therapies—cisplatin via DNA cross-linking and paclitaxel via microtubule stabilization—which function independently of E2F2-mediated pathways that regulate cell cycle and immune checkpoints.

E2F2’s therapeutic relevance is supported by three lines of evidence: (1) positive correlations between its expression and sensitivity to alternative chemotherapeutics (e.g., crizotinib and erlotinib) and targeted agents (e.g., ruxolitinib); (2) significant associations between low E2F2 levels and favorable anti-CTLA4 responses in IPS analyses; (3) regulatory connections to established immunotherapy biomarkers (PD1 and CTLA4). These findings indicate context-dependent utility, as E2F2 does not predict platinum/paclitaxel responses but may inform decisions on alternative systemic therapies and immunotherapies. Further validation in clinical cohorts is needed to confirm these translational implications, emphasizing the importance of subtype-specific and context-aware interpretation of E2F2’s therapeutic potential in SOC.

Given the demonstrated prognostic significance of E2F2, its association with immune checkpoint expression, and links to drug sensitivity, exploring potential therapeutic strategies targeting E2F2 holds substantial translational relevance.

In terms of small-molecule inhibitors, current research has made progress in developing agents that target E2F transcriptional activity or its protein–protein interactions with retinoblastoma (Rb) family members (Konagaya et al., 2024; Bockus et al., 2025). While E2F2-selective inhibitors remain in early developmental stages (Chen et al., 2023), preclinical studies on pan-E2F inhibitors have shown promise in suppressing tumor growth by disrupting cell-cycle progression. This aligns with our findings that E2F2 co-expresses with cell-cycle-related genes (e.g., ORC1 and RAD54L) and promotes metastatic potential *in vitro*, supporting the rationale for such inhibitory approaches.

Combination strategies also warrant consideration. Our results indicating correlations between E2F2 expression and sensitivity to chemotherapeutics (e.g., crizotinib and erlotinib) as well as immune checkpoint molecules (e.g., PD1 and CTLA4) suggest potential synergies. For example, inhibiting E2F2 may enhance responses to anti-CTLA4 therapy in patients with high E2F2 expression, a possibility supported by our IPS analysis revealing differential responses based on E2F2 levels.

Additionally, gene silencing approaches such as RNA interference (RNAi) or CRISPR-based strategies represent viable avenues. Our *in vitro* experiments, where E2F2 knockdown reduced cell migration and invasion, provide preliminary evidence for the feasibility of direct E2F2 inhibition in suppressing serous ovarian carcinoma (SOC) progression.

It is important to acknowledge that these therapeutic strategies require extensive preclinical validation. Nevertheless, the collective findings presented herein highlight E2F2 as a promising target, with further research in this direction potentially yielding novel therapeutic options for SOC.

We acknowledge certain limitations in our study. Regarding the functional validation of E2F2, while SKOV3 (a well-characterized SOC model) was selected as the primary system for its relevance to our focus on the serous subtype, and A2780 served as a supplementary model due to its robust E2F2 expression, we recognize that A2780 does not fully align with the serous subtype specificity central to our investigation. Nevertheless, consistent functional phenotypes observed in both cell lines provide complementary evidence that support hypotheses derived from SOC-focused analyses, and this concordance across lines, despite subtype differences, strengthens the reliability of our findings on E2F2’s oncogenic roles in ovarian carcinogenesis. Furthermore, our study primarily relied on bioinformatic analyses and *in vitro* experiments, necessitating further *in vivo* validation. Additionally, the validation of our findings in larger cohorts is required to enhance their robustness.

## Conclusion

The significance and novelty of this study lie in the integrated analysis across multiple databases to characterize the relationship between E2F2 and serous ovarian carcinomas (SOCs), as well as to investigate its potential role in guiding SOC treatment. High E2F2 expression indicates poor prognosis in patients with SOCs, is associated with immune cell infiltration, and serves as an independent risk factor. It may act as a potential biomarker for disease diagnosis, patient stratification, and guidance of personalized therapy.

## Data Availability

The original contributions presented in the study are included in the article/Supplementary Material; further inquiries can be directed to the corresponding authors.

## Abbreviations

DEGs, differentially expressed genes; HGSOCs, high-grade serous ovarian cancers; IC_50_, half-maximal inhibitory concentration; OS, overall survival; PFS, progression-free survival; SOCs, serous ovarian cancers; Tregs, regulatory T cells.
