# Supplementary figures and images for: Integrated analysis and experimental validation of E2F2 as a potential prognostic biomarker and its oncogenic roles in serous ovarian cancer
Source: Front Mol Biosci. 2025 Sep 23;12:1661558. doi: 10.3389/fmolb.2025.1661558 (PMC12501506; doi:10.3389/fmolb.2025.1661558)

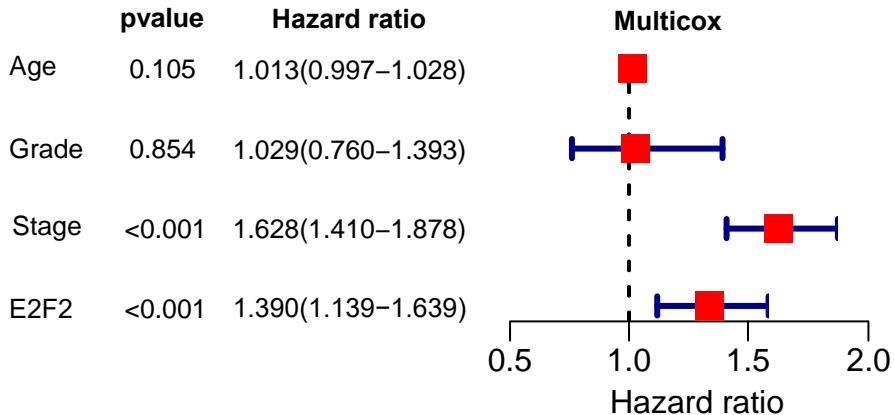

Supplement: Supplementary file 1 [file Image2.pdf]

|       | <b>pvalue</b> | <b>Hazard ratio</b> |
|-------|---------------|---------------------|
| Age   | 0.363         | 1.007(0.992–1.023)  |
| Grade | 0.630         | 1.075(0.800–1.446)  |
| Stage | <0.001        | 1.625(1.412–1.869)  |
| E2F2  | <0.001        | 1.370(1.142–1.599)  |

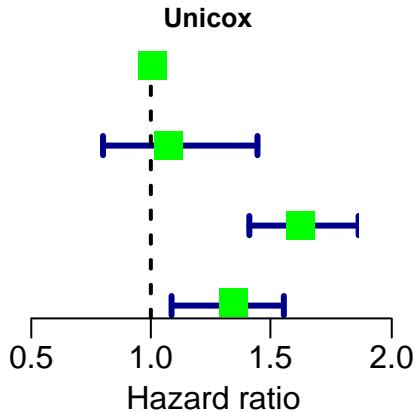

Supplement: Supplementary file 2 [file Image1.pdf]
